# Molecular Insights Reveal *Psy1*, *SGR,* and *SlMYB12* Genes are Associated with Diverse Fruit Color Pigments in Tomato (*Solanum lycopersicum* L.)

**DOI:** 10.3390/molecules22122180

**Published:** 2017-12-08

**Authors:** Song-I. Kang, Indeok Hwang, Gayatri Goswami, Hee-Jeong Jung, Ujjal Kumar Nath, Hee-Ju Yoo, Je Min Lee, Ill Sup Nou

**Affiliations:** 1 Department of Horticulture, Sunchon National University, 255 Jungangro, Suncheon, Jeonnam 57922, Korea; 1160041@scnu.ac.kr (S.-I.K.); usename@hanmail.net (I.H.); gayatri_bau@yahoo.com (G.G.); gml79wjd@sunchon.ac.kr (H.-J.J.); ujjalnath@gmail.com (U.K.N.); 2 Department of Horticultural Science, Kyungpook National University, 80 Daehakro, Bukgu, Daegu 41566, Korea; yhj901003@knu.ac.kr (H.-J.Y.); jemin@knu.ac.kr (J.M.L.)

**Keywords:** carotenoid, flavonoid, chlorophyll, gene mutation, fruit color, tomato

## Abstract

The color of tomato (*Solanum lycopersicum*) fruit flesh is often used as an indicator of quality. Generally, fruit color is determined by the accumulation of carotenoids and flavonoids, along with concomitant degradation of chlorophylls during ripening. Several genes, such as *phytoenesynthetase1* (*Psy1*), *STAY-GREEN* (*SGR*), and *SlMYB12*, have been extensively studied to elucidate the genes controlling fruit coloration. In this study, we observed low carotenoid levels without degradation of chlorophylls in green-fruited tomato caused by mutations in three genes, *Psy1*, *SGR*, and *SlMYB12*. We crossed two inbred lines, BUC30 (green-fruited) and KNR3 (red-fruited), to confirm the causal effects of these mutations on fruit coloration. The F_2_ population segregated for eight different fruit colors in the proportions expected for three pairs of gene, as confirmed by a chi-square test. Therefore, we developed a population of tomato with diverse fruit colors and used molecular markers to detect the genes responsible for the individual fruit colors. These newly-designed DNA-based markers can be used for selecting desired fruit color genotypes within adapted breeding materials and cultivars for breeding.

## 1. Introduction

Pigments are mainly produced by plants in their reproductive parts (flowers and fruits) to attract pollinators and seed dispersers. Color is considered one of the most important quality attributes of tomato fruit for consumer acceptance [1]. The color of flowers and fruits of plants mainly result from the accumulation of carotenoid and flavonoid pigments [2]. The red color of ripe tomato fruit results from the accumulation of the carotenoid *trans*-lycopene during ripening. Apart from lycopene, tomato fruit also contains β-carotene, phytoene, violaxanthin, and lutein. Tomato fruit can be orange or yellow due to mutations of carotenoid pathway genes [3].

Tomato fruit peels also accumulate flavonoids, which are yellow-colored naringeninchalcones. Flavonoids are accumulated in the cuticle and are responsible for the yellow color in the peel at the breaker stage [4]. Flavonoids are a group of polyphenols, which can be grouped into different classes based on their core structures: chalcones, flavanones, aurones, flavonols, and anthocyanins. A wide range of flavonoids (>6000) have been identified in nature [2]. This diversity may be due to modification by different enzymes, such as glycosyl, malonyl, acyl, and methyl transferases. Apart from ascribing color to plant tissues, flavonoids are also involved in many other aspects of plant growth and development, such as resistance against pathogens and protection from ultraviolet light [5,6]. Flavonoids are also hydrophilic antioxidants and are recognized as health-promoting components [7,8,9,10,11]. Anthocyanins produce red, purple, and blue pigments in flowers and fruits, chalcones and aurones produce yellow pigments, and flanovols produce co-pigments [12,13]. In addition, the flavonols quercetin-3-rutinoside (rutin) and kaempferol-3-rutinoside are found in the peel of ripened tomato fruit [14,15]. However, mutations of carotenoid and flavonoid biosynthesis pathways and transcriptional regulation of the genes can produce different types of fruit color. 

Ripe tomatoes have a wide range of colors, like red, orange, pink, yellow, brown, green, purple, black, and even white. The color of tomato fruit during ripening is also associated with concomitant degradation of chlorophylls [12,13]. Carotenoid and flavonoid biosynthesis and chlorophyll degradation pathways are closely related to the color of tomato fruit. A lack of chlorophyll degradation with a high accumulation of lycopene leads to brown-colored fruits. Naringeninchalcone (NC) is an important flavonoid accumulated mainly in the fruit peel during ripening. The transparent fruit peel observed in pink tomatoes is caused by a lack of NC [16].

Genes and enzymes involved in carotenoid biosynthesis have been extensively studied to better understand the mechanisms of fruit coloration [17]. Molecular markers have been designed to tag the genes responsible for the different colors in tomato fruit, including *Psy1*, *CRTISO*, *CYC-B*, *SGR*, and *SlMYB12* [18]. *Psy1* encodes phytoene synthase, which is involved in the formation of 15-*cis*-phytoene by the condensation of two molecules of geranylgeranyl diphosphate, which is the first condensation step of the carotenoid biosynthesis pathway. The knock-out mutation of *Psy1* results in lower accumulation of carotenoids, which confers yellow-colored flesh [19,20]. The fruit color of *tangerine* mutants, such as *tangerine^mic^* and *tangerine*^3183^, results from loss of function of *CRTISO*, which encodes a carotenoid isomerase necessary for the production of *trans*-lycopene. In the absence of the isomerase, prolycopene accumulates, resulting in an orange color instead of red [21,22,23]. *CYC-B* encodes lycopene β-cyclase, which converts lycopene to β-carotene. The amino acid sequence of lycopene β-cyclaseis similar to that of capsanthin-capsorubin synthase, which is responsible for red xanthophylls in pepper fruit (*Capsicum annum* [24]).The *old gold* (*og*) and *old-gold crimson* (*og^C^*) null mutations in *CYC-B* result in decreased β-carotene and increased lycopene biosynthesis, which confers a change in fruit color from yellow to deep red [24]. We previously reported four Single Nucleotide Polymorphsims (SNPs) in the promoter region of *CYC-B* that are associated with the orange fruit color [25] in tomato. A mutation in *STAY-GREEN* (*SGR*) inhibits chlorophyll degradation during ripening, and when accompanied by an accumulation of lycopene, this leads to brown-colored fruit [26,27]. *SGR* positively regulates *ripening-inhibitor* (*RIN*), which plays a critical role in the regulation of chlorophyll degradation in tomato leaves and fruit [28,29]. *SlMYB12* is a transcriptional regulator of NC biosynthesis and is considered a candidate gene for the pink-fruit trait in tomato. Overall, tomato fruit color is determined by the accumulation of intermediate products of carotenoid and flavonoid biosynthesis and chlorophyll degradation.

In this study, we aimed to identify the mutations in *Psy1*, *SGR*, and *SlMYB12* that are responsible for many of the different fruit color phenotypes in tomatoes and also to design a set of molecular markers associated with the genes corresponding to tomato fruit color.

## 2. Materials and Methods

### 2.1. Plant Materials and Genomic DNA Extraction

**Figure 1:**
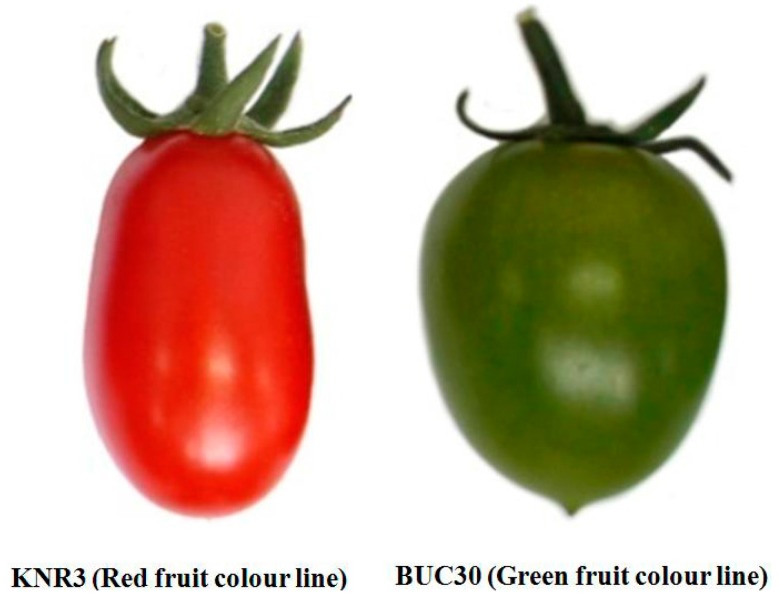
Comparison of fruit pigmentation in the red-fruited line (KNR3) and the green-fruited line (BUC30) at the completely-ripened stage achieved 57 days after pollination.

Seeds of two tomato inbred lines, KNR3 (red color when ripe) and BUC30 (green color when ripe), were collected from the Kana Seed Co. Ltd. and Bunong Seed Co. Ltd. Seoul, Korea, respectively (Figure 1). The two inbred lines were crossed and the F_2_ population of 494 individuals was developed by self-pollinating the F_1_ plants. Genomic DNA of the parental lines and the F_2_ population was extracted from eight-week-old leaf tissue using the DNeasy Plant Mini Kit from Qiagen (Germantown, MD, USA).

### 2.2. PCR Amplification

All 494 F_2_ individuals and the parental lines were screened for mutations using PCR. PCR was carried out in a 20 μL reaction containing 1× buffer, 2.0 mM MgCl_2_, 0.2 mM dNTPs, 0.2 μM primers, 5 ng template DNA, and 0.5 units Taq polymerase (Promega, Madison, WI, USA). The PCR was performed with the following conditions: initial denaturation at 94°C for 5 min followed by 30 cycles of denaturation at 94°C for 30 s, annealing at 60°C for 30 s, and extension at 72 °C for 45 s, and a final elongation at 72°C for 5 min. The PCR products were loaded into 1.5% agarose gels containing Tris-boric acid EDTA (TBE) buffer and separated by electrophoresis at 80 volts for 1 h before visualization under ultraviolet (UV) light.

### 2.3. RNA Isolation and cDNA Synthesis

Total RNA was extracted from leaf samples using an RNeasy mini kit (Qiagen, Germantown, MD, USA) following the manufacturer’s guide. DNA contamination was removed using an RNase-free DNase (Promega, Madison, WI, USA) treatment following the manufacturer’s protocol. The extracted RNA was quantified by UV spectrophotometry at A260 using a NanoDropND-1000 and NanoDrop v3.7 software (NanoDrop Technologies, Wilmington, DE, USA). Complementary DNA (cDNA) was synthesized from 5 μg of RNA from all samples using the Superscript III First Strand cDNA synthesis Kit (Invitrogen, Tokyo, Japan) in a 20-μL reaction per the manufacturer’s protocol and utilized for PCR amplification. The cDNA was stored at −20 °C until use. 

### 2.4. Cloning and Sequencing 

The gene sequence and coding sequence (CDS) were amplified from genomic DNA and cDNA, respectively, by polymerase chain reaction (PCR) using pfuTurbo DNA polymerase (Stratagene, Agilent Technologies, Santa Clara, CA, USA). PCR was carried out using the primers (Supplementary Table S1) that were designed based on the sequence information from the Sol genomics network [30]. The PCR product was introduced into a blunt cloning vector using the Topcloner Blunt kit (Enzynomics, EZ012M, Daejeon, Korea). The ligation products were transformed into *Escherichia coli* DH5-alpha competent cells. The recombinant plasmids were purified using the Plasmid Mini kit (Qiagen, 12125, Germantown, MD, USA) and sequenced at Macrogen Corp (Rockville, MD, Korea). Three colonies were sequenced as biological replicates.

### 2.5. Detection of SNPs Using Hybprobe

High-resolution melting (HRM) analysis was conducted using 3′-blocked and unlabeled oligonucleotide probes (Hybprobe) to detect SNPs. PCR was performed using the saturating dye LC Green Plus (Roche, Basel, Switzerland) to generate melting curves corresponding to the genotypes. Melting curves were generated and analyzed using the LightScanner Instrument System (Supplementary Figure S1). PCR was performed as follows: pre-denaturation at 95 °C for 5 min followed by 45 cycles of denaturation at 95 °C for 20 s, annealing at 60 °C for 20 s, and extension at 72 °C for 30 s, and with a final extension at 72 °C for 40 s. The primer and probe sets for SNP detection are described in Supplementary Table S1.

### 2.6. HPLC Analysis

Three plants were randomly selected from each group of fruit color phenotypes (eight in total) in the 494 F_2_ individuals and the parental lines. Five ripe fruit from each selected plant were used for biochemical analysis using high performance liquid chromatography (HPLC). The phytoene, carotenoids, and chlorophylls were separated using reverse-phase columns (Kinetex 2.6 μm, C18 100A, 100  ×  4.60 mm, Phenomenex, Torrance, CA, USA). The whole-fruit extracts were filtered with a 0.2 μm PTFE filter prior to injection. The mobile phase consisted of two solvents, A = 78% methanol and B = 100% ethyl acetate. The carotenoid and chlorophyll analyses were performed using a 1260 Infinity series HPLC instrument (Agilent, Santa Clara, CA, USA) and Chemstation software (Santa Clara, CA, USA). The carotenoids and chlorophylls were identified and quantified based on their retention times and the absorbance between 280 nm and 480nm for carotenoids and 660nm for chlorophylls following the protocol described by Yoo et al. [31]. The values represented in this study are the mean of three biological replicates.

### 2.7. Statistical Analysis

The biochemical contents in the different colored fruit phenotypes were analyzed using one-way ANOVA and Tukey’s pairwise comparison in MINITAB v.17 (State College, PA, USA) statistical package. The biochemical contents are presented as mean weight ± SD. Standardized data (i.e., the mean subtracted from the case and the result divided by the standard deviation) were used for principal component analysis (PCA). Pigment contents of the eight different fruit color phenotypes analyzed by HPLC at the ripening stage were set as variables using MINITAB v.17 statistical package (Minitab Inc., State College, PA, USA).

## 3. Results and Discussion

### 3.1. Mutation of the RIN Gene and Its Effect on Tomato Color

To confirm the molecular inheritance of green-fruited tomatoes, we collected commercial red- and green-fruited tomatoes. Green tomatoes are green at maturity (Figure 1). The ripe green tomatoes of the inbred line BUC30 used in this study were sweet, similar to the ripe red tomatoes of the KNR3 line, and the ripening stages were similar among both lines. Normal tomato fruit turn red during ripening, which is regulated by the transcription factor encoded by *RIN*. Therefore, a mutation in *RIN* may be responsible for the green coloration in ripe fruit. The *RIN* mutation has pleiotropic effects on ripening, resulting in abnormal and extremely delayed ripening [32]. The *RIN* mutant fails to attain a normal level of pigmentation as a result of decreased accumulation of carotenoids, particularly lycopene, as well as reduced chlorophyll degradation [33]; therefore, the fruit of these tomatoes remain green compared to the wild-type red fruits. Fruit of the *RIN* mutant eventually turn lemon yellow in color after several weeks, but lack the normal flavor or aroma, possibly due to failure of true ripening [32,33,34]. We investigated the *RIN* gene in our green-colored tomatoes by designing primers at different positions of the gene covering the 3′-UTR to the 5′-UTR, but failed to detect any mutation (data not shown). Therefore, we concluded that our green tomatoes carried the wild-type *RIN* gene.

### 3.2. Mutation of the Psy1 Gene and Its Effect on Tomato Color

**Figure 2:**
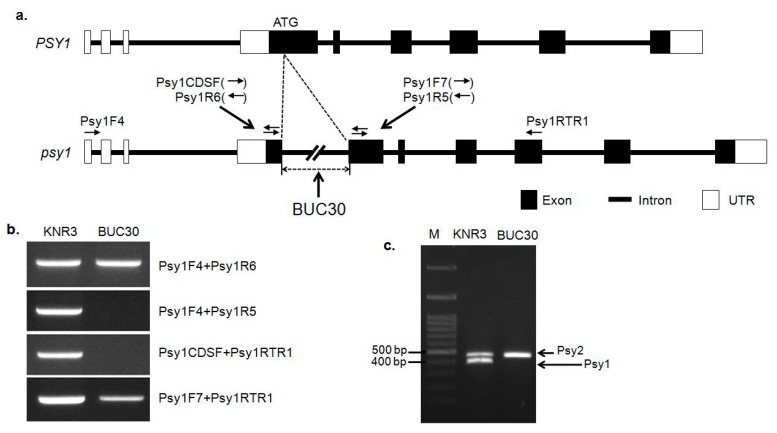
Gene structures of the wild-type and mutated forms of *Psy1* and their amplification products using different sets of primers designed at different positions of the gene to detect mutations. (**a**) Representation of the putative long insertion in *Psy1*, which causes the green fruit in BUC30 (lower panel) compared to normal *Psy1* in the red-fruited line; (**b**) PCR amplification of *Psy1* from the individual inbred lines with different primers sets. The primers sets include: 5′-UTR forward (Psy1F4), exon 1 reverse (Psy1R5 and Psy1R6), exon 1 forward (Psy1CDSF and Psy1F7), and exon 4 reverse (Psy1RTR1). Amplicons were seen in the red-fruited inbred line (KNR3) when the Psy1F4 + Psy1R6, Psy1F4 + Psy1R5, Psy1CDSF + Psy1RTR1, and Psy1F7 + Psy1RTR1 primer sets were used. However, in the green-fruited inbred line (BUC30), the fragments were not amplified when the Psy1F4 + Psy1R5 and Psy1CDSF + Psy1RTR1 primer sets were used, while PCR products of the expected size were amplified with the Psy1F4 + Psy1R6 and Psy1F7 + Psy1RTR1 primer sets; (**c**) PCR products amplified from the individual inbred lines with the primer set Psy1CDSF + Psy1R5. Two amplicons were produced in the KNR3 line where the upper band belongs to *Psy2* and the lower band belongs to *Psy1*. The *Psy1* band was not detected in the green-fruited BUC30 line. M: 100 bp size marker.

The mutation in *Psy1* results in a low accumulation of carotenoids and a yellow-flesh phenotype in tomato fruit [19,20]. Therefore, this lack of carotenoid accumulation during the ripening process may be responsible for the green-fruited phenotype at the ripening stage. To further investigate the molecular mechanisms of the green peel coloration in ripe tomatoes, we analyzed the structure of *Psy1*. The *Psy1* gene in the inbred lines KNR3 and BUC30 was amplified using the following primers: exon 1 forward (Psy1F7 and Psy1CDSF), exon 4 reverse (Psy1RTR1), 5′-UTR forward (Psy1F4), and exon 1 reverse (Psy1R5 and Psy1R6) (Figure 2a and Supplementary Table S1). However, only the primer sets Psy1F4 and Psy1R6, and Psy1F7 and Psy1RTR1 amplified the expected PCR product size in KNR3 (red-fruited line). No products were amplified in BUC30 (green-fruited line) with the primer sets Psy1F4 and Psy1R5, and Psy1CDSF and Psy1RTR1, indicating that there might be a large insertion in the first exon (Figure 2a,b). To confirm this result, we conducted PCR using a different primer set (Psy1CDSF and Psy1R5) designed as an insertion/deletion (InDel) marker, with which two fragments were amplified in KNR3 and one fragment in BUC30 (Figure 2c). Cloning, sequencing, and alignment of the PCR products confirmed that the upper band was the *Psy2* fragment, while the lower band was the *Psy1* fragment. *Psy1* was not amplified in BUC30 (green-fruited line), strengthening our hypothesis of a long insertion in the first exon of *Psy1* in BUC30 (Figure 2a,c). This mutation may cause a loss of function of *Psy1* due to aberrant splicing of its transcripts, resulting in low carotenoid accumulation in tomato fruit.

### 3.3. Mutation in the SGR Gene and Its Effect on Tomato Color

**Figure 3:**
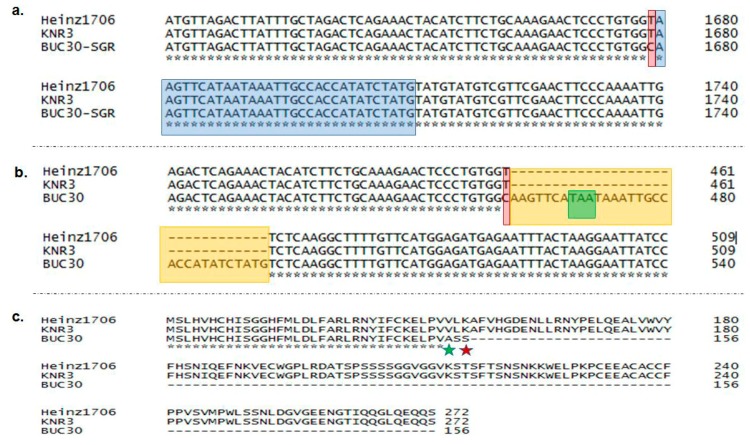

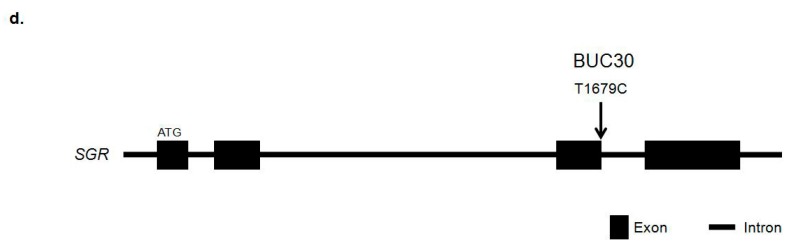
Alignment of DNA and protein sequences, and the position of the mutation of the *SGR* gene. (**a**) Sequence alignment of *SGR* amplified from genomic DNA of the red-fruited line (KNR3) and the green-fruited line (BUC30) compared with the reference genotype Heinz1706, showing a 1-bp substitution at position 1679 bp (highlighted in red). Alternative splicing was found in the following 31 base pairs highlighted in blue, which was confirmed in the CDS of BUC30; (**b**) Sequence alignment of *SGR* cDNA of the red-fruited line (KNR3) and the green-fruited line (BUC30) compared with the reference genotype Heinz1706, showing a 1-bp alteration at position 461 bp (highlighted in red) followed by a 31-bp insertion, which resulted in alternative splicing in the mRNA due to the conversion of the intron to an exon (the orange box). This insertion resulted in an early stop codon (green box inside the orange box); (**c**)Amino acid alignment showing one amino acid substitution (green star) due to the non-synonymous SNP and termination of translation (red star) of the protein in the green-fruited line BUC30; (**d**) Graphical representation of the *SGR* gene based on the genomic sequence, where the T to C substitution is positioned at the end of the third exon.

In general, a sharp decrease in the content of chlorophylls and a concomitant increase in the synthesis of carotenoids occur during tomato ripening. Therefore, green-colored fruit may occur due to low contents of carotenoids and less degradation of chlorophylls during ripening. Barry et al. [27] found that a mutation in *SGR* results in reduced chlorophyll degradation during ripening. We hypothesized that both the *Psy1* and *SGR* mutations may be involved in the green fruit color in BUC30. To explore this, we cloned and sequenced the *SGR* cDNA from BUC30. A 1-bp substitution (T to C) at position 461 bp and a 31-bp insertion were found in the green-fruited line compared to the red-fruited lines KNR3 and the reference line Heinz1706 (the inbred cultivar used to develop the tomato genomic database [30]). We also cloned and sequenced *SGR* from the genomic DNA of BUC30 and found a non-synonymous SNP with a 1-bp substitution (T to C) at position 1679 bp. This mutation occurred at the last base pair of exon 3, which resulted in a 31-bp conversion of an intron to an exon in the CDS (Figure 3a,b). This conversion from an intron to an exon might cause alternate splicing of the mRNA, which was detected in the green-fruited tomato line via sequence alignment of *SGR* cDNA with that of KNR3 and the reference line Heinz1706 (Figure 3b). The non-synonymous SNP with a 1-bp substitution (T to C) in *SGR* of BUC30 caused alternate splicing in the *SGR* mRNA and an amino acid change from valine to alanine, which resulted in an early stop codon (depicted as a green star in Figure 3c and Supplementary Data 1). These two mutations (the SNP and the insertion) resulted in loss of function of *SGR*. The mutations in *SGR* from BUC30 were also confirmed by 3′-blocked and unlabeled oligonucleotide probing with BUC30 genomic DNA using the HybProbe method. Two different melting curves were distinguishable for the two different sequences in KNR3 and BUC30 (Supplementary Figure S1). Alternative splicing in genes related to carotenoid, chlorophyll, and flavonoid biosynthesis and metabolism was reported in kiwi fruit [35] and in wild barley (*Hordeum chilense*; [36]).

### 3.4. Mutation of the SlMYB12 Gene and Its Effect on Tomato Fruit Color

*SlMYB12* is a transcription factor that regulates flavonoid biosynthesis in tomato fruit during ripening and also the color of the flesh and peel of the fruit. A strong correlation between the expression of *SlMYB12* and the level of carotenoids in tomato fruit was reported by Veerappan et al. [37]. Three allelic variants of *SlMYB12*, a 603-bp deletion, a nucleotide substitution (C to T), and a 1-bp insertion (TG to TAG) have been reported as being responsible for the pink fruit color in tomato [19]. To elucidate the roles of these mutations on green fruit color in tomatoes, two independent primer sets (aF1/aR6 and aF1/aR5) were used as insertion/deletion (InDel) markers (Supplementary Table S1) for separate PCR reactions, then the PCR products were mixed and separated on a 1.5% agarose gel. The presence of twobands (950 bp and 614 bp) were observed in the KNR3 line, but both of the bands were absent in BUC30, indicating that *SlMYB12* was present in KNR3 (red-fruited line), but had been deleted in BUC30 (green-fruited line). By contrast, a single band of 347 bp was detected in BUC30, suggesting that a 603-bp deletion of *SlMYB12* (Supplementary Figure S2) may also be responsible for the green fruit color in BUC30. The deletion was hypothesized to cause transcriptional repression of *SlMYB1*2 in tomato [38]. Based on the results of this study, we concluded that the mutations of *Psy1*, *SGR*, and *SlMYB12* are present in the BUC30 line at ripening. 

### 3.5. Characterization of the Psy1, SGR, and SlMYB12 Mutations in the F_2_Segregants

To follow the segregation of the *Psy1*, *SGR*, and *SlMYB12* mutations, a segregating F_2_ population of 494 individuals was developed by crossing two inbred lines, KNR3 (red-fruited line) and BUC30 (green-fruited line). The F_2_ generation exhibited eight different fruit color phenotypes: red, yellow, brown, pink, light yellow, pink brown, yellow green, and green, following the expected ratio of 27:9:9:9:3:3:3:1 for threesegregating gene pairs (chi-square value = 5.9^ns^; *p* < 0.05) (Supplementary Figure S3 and Supplementary Table S2). The fruit color of the F_1_ population was the normal red color. The phenotypes in the F_2_segregants were produced due to different levels of accumulation of carotenoids and chlorophylls, which are regulated by three genes, *Psy1*, *SGR*, and *SlMYB12* (Supplementary Table S3). The HPLC analysis of the eight different phenotypes for carotenoid and chlorophyll content and peel color further illustrated the presence of the mutations (Supplementary Table S4).

**Figure 4:**
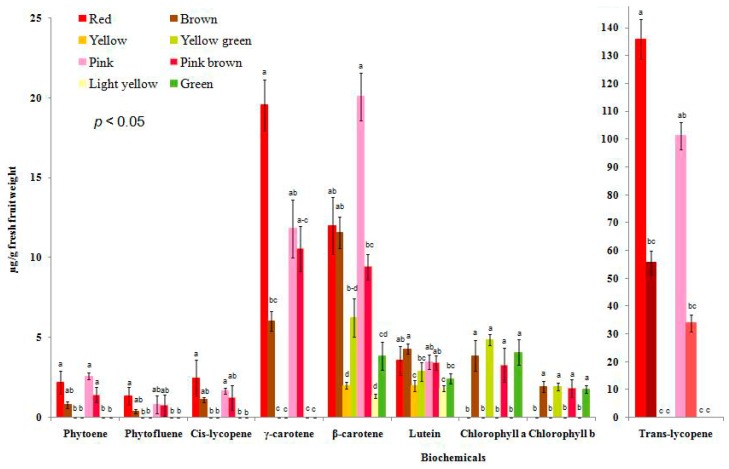
Biochemical profiles of the eight different fruit color phenotypes in the F_2_ population of the cross between the red-fruited line (KNR3) and the green-fruited line (BUC30). Values indicate the mean of the HPLC results of five fruit harvested from three randomly-selected plants for each fruit color 57 days after pollination. Error bars are the standard deviation (SD) values of the HPLC results of the respective fruit color. Different letters indicate significance differences among the fruit phenotypes for the particular biochemicals tested at the *p* < 0.05 level.

In the green fruit phenotype, most of the carotenoids were absent except for β-carotene and lutein, but chlorophyll a and chlorophyll b were present (Figure 4). The presence of chlorophylls and the transparent peel in the green fruits confirmed the failure of chlorophyll degradation (Supplementary Table S4) and the absence of carotenoids. We detected the mutated *Psy1*, *SGR*, and *SlMYB12* genes in the green-fruited F_2_segregants (Table 1, Figure 2b,c and Supplementary Figures S1 and S2) using the combinations of primer pairs shown in Supplementary Table S1. In the red-fruited plants, a significantly higher amount of *trans*-lycopene was present followed by γ-carotene, β-carotene, and lutein, and a complete absence of chlorophylls compared to the green-fruited plants (Figure 4). This result indicated that a normal accumulation of carotenoids and simultaneous degradation of chlorophylls occurred in the red-fruited plants, suggesting that the wild-type *Psy1*, *SGR*, and *SlMYB12* genes were present (Table 1 and Supplementary Table S4), which was in accordance with the findings of Davies [12] and Tanaka et al. [13]. The pink-fruited tomatoes contained a significant higher amount of *trans*-lycopene followed by β-carotene and γ-carotene, and lacked chlorophylls compared to green fruited one (Figure 4 and Supplementary Table S4), which was also supported by previous reports [2,16,37].

Higher amounts of *trans*-lycopene, β-carotene, γ-carotene, and chlorophylls were found in the brown-fruited tomatoes compared to the green-fruited plants due to the presence of wild-type *Psy1* and *SlMYB12* coupled with mutated *SGR* (Figure 4, Table 1 and Supplementary Table S4). The yellow fruit phenotype was produced due to the combination of mutated *Psy1* with wild-type *SGR* and *SlMYB12*. *Psy1* catalyzes the first step in carotenoid biosynthesis; therefore, the fruit failed to accumulate lycopene (Supplementary Table S4) as suggested by Fray and Grierson [39]. In the light-yellow fruit phenotype, the presence of mutated *Psy1* and *SlMYB12* was found. By contrast, mutated *Psy1* and *SGR* were confirmed in the yellow-green phenotype. In the pink-brown phenotype, mutated *SGR* and *SlMYB12* were detected along with wild-type *Psy1* (Table 1).

**Figure 5:**
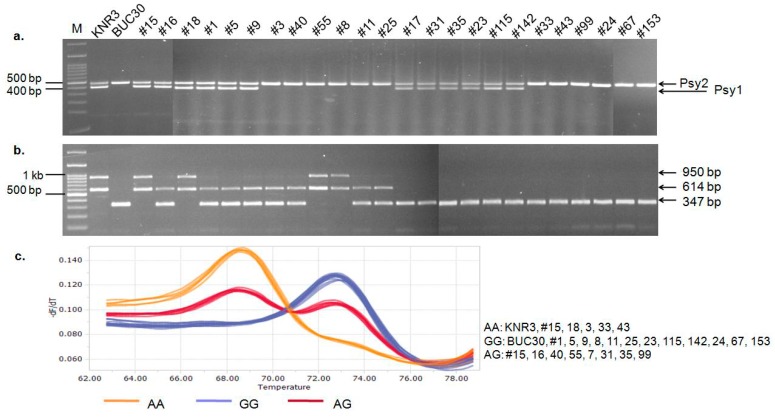
Validation of the molecular markers designed to follow the mutations of *Psy1*, *SlMYB12*, and *SGR* using randomly-selected plants from an F_2_ population segregating for eight fruit color phenotypes. (**a**) PCR amplicons of mutated *Psy1* using Psy1CDSF+Psy1R5 primers; (**b**) PCR amplicons of mutated *SlMYB12* using MYB12-603del-aF1+aR6 and MYB12-603del-aF1+aR5 primers; (**c**) High-resolution melting (HRM) curve of the 1-bp substitution in *SGR* using 3′-blocked and unlabeled oligonucleotide probes (Hybprobe-complementary sequence). #15, #16, and #18 (red-fruited plants); #1, #5, and #9 (brown-fruited plants); #3, #40, and #55 (yellow-fruited plants); #8, #11, and #25 (yellow-green-fruited plants); #17, #31, and #35 (pink-fruited plants); #23, #115, and #142 (pink-brown-fruited plants); #33, #43, and #99 (light-yellow-fruited plants); #24, #67, and #153 (green-fruited plants); and M is the 100-bp molecular ladder.

Our results indicated that variable levels of carotenoids and chlorophylls accumulated in the different fruit color phenotypes, which were directly associated with different combinations of mutated and wild-type *Psy1*, *SGR*, and *SlMYB12* genes. The nucleotide diversity of genes influences the carotenoid levels in fruit during ripening by regulating biosynthesis or signaling pathways [17]. All of the 494 F_2_ individuals segregating for the eight different fruit color phenotypes were screened with the markers designed in this study, listed in Table 2, to correlate the mutations of *Psy1*, *SGR*, and *SlMYB12* with a particular fruit color phenotype. All of our markers were 100% effective at predicting the phenotype (see an example in Figure 5a–c).

### 3.6. Association among Biochemical Accumulation, Gene Mutation, and Different Fruit Colors

Principal component analysis of the contents of nine individual biochemicals (carotenoids and chlorophylls) and the mutated or wild-type form of *Psy1*, *SGR*, and *SlMYB12* showed a major contrast between the accumulation of individual biochemicals and gene mutations towards diverse fruit color phenotypes in tomato. The first two principal components (PCs) explained 90.2% of the total variation in the data (Table 3). PC1 accounted for 66% of the total variation, which was largely influenced by higher positive coefficients for *trans*-lycopene, γ-carotene, *cis*-lycopene, phtoene, phytofluene, β-carotene, and lutein versus lower negative coefficients for chlorophyll a and chlorophyll b (Table 3).

**Figure 6:**
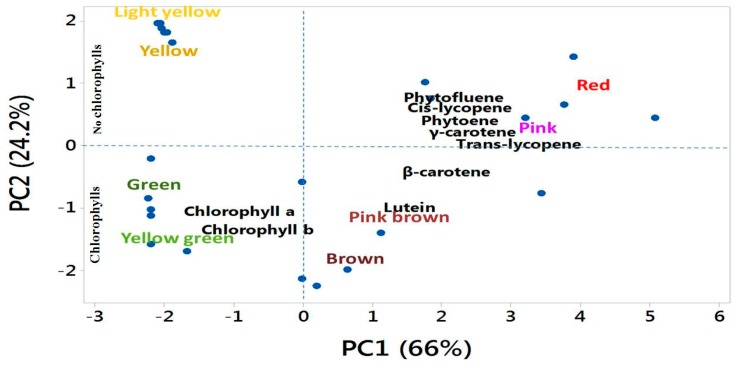
Biplot of biochemical content relevant to the tomato fruit color in the eight different fruit-color phenotypes as determined by principal component analysis (PCA).

In PC1, *trans*-lycopene, γ-carotene, *cis*-lycopene, phtoene, and chlorophyll a, and in PC2, phytofluene, chlorophyll b, chlorophyll a, and lutein explained the majority of the variation of the entire dataset (Table 3). PC1 clearly separated the red and pink phenotypes from the green and yellow green phenotypes to the furthest opposite diagonal quadrant in the PCA biplot (Figure 6). This was also evident from the higher mean PC scores in opposite directions in these fruit color (red and pink +3.59 and +2.90, respectively, and green and yellow-green −2.20 and −2.04, respectively) phenotypes (Table 3). The red and pink fruit-color phenotypes were associated with complete degradation of the chlorophyll in the fruit. In contrast, green and yellow-green fruit-color phenotypes were associated with a lack of chlorophyll degradation coupled with no accumulation of carotenoids (Supplementary Table S4). PC2 explained 24.2% of the variation in the data, which was largely dominated by higher coefficients of phytofluene and phytoene and lower coefficients of chlorophyll b, chlorophyll a, lutein, and β-carotene (Table 3).

PC2 also clearly separated the yellow and light-yellow from the brown and pink-brown phenotypes to the furthest opposite diagonal quadrant in the PCA biplot (Figure 6) and by their contrasting PC scores (Table 3). This result further illustrated that the yellow and light-yellow phenotypes are associated with complete degradation of chlorophylls with a slight accumulation of β-carotenoid and lutein, whereas the brown and pink-brown phenotypes are associated with the accumulation of chlorophylls with different levels of carotenoids (Supplementary Table S4).

## 4. Conclusions

In this study, we confirmed the association of *Psy1*, *SGR*, and *SlMYB12* genes and their mutations with tomato fruit color phenotypes based on catalyzing the breakdown or accumulation of different carotenoids and chlorophylls. In ripe tomato fruit of the KNR3 line (red-fruited line), lycopene accounted for approximately 76% of the pigment, with the remaining pigments mainly γ-carotene, β-carotene, and phytoene, and complete degradation of chlorophylls. This line contained the wild-type forms of *Psy1*, *SGR*, and *SlMYB12*. By contrast, we found that the green-fruited line, BUC30, contained the mutated form of each of these genes. This plant contained no carotenoids and there was no degradation of chlorophylls. The mutant form of *SGR* and a lack degradation of chlorophylls were associated with the brown-fruited phenotype. The pink fruit-color phenotype was due to the mutated form of *SlMYB12*, and the yellow phenotype resulted from a mutation in *Psy1*. In addition, the light-yellow phenotype carried the mutated form of *Psy1* and *SlMYB12*, the pink-brown phenotype contained the mutated form of *SGR* and *SlMYB12*, and the yellow-green phenotype was associated with the *Psy1* and *SGR* mutations. Additionally, our study provided new molecular markers for identifying the eight different fruit color phenotypes observed in the F_2_ population. The molecular markers designed and identified in this study may be useful in breeding to develop and select desired fruit-color phenotypes in tomato.

## Figures and Tables

**Table 1:** Summary of the genetic makeup of the different fruit color phenotypes in the F_2_ population for *Psy1*, *SGR*, and *SlMYB12* in tomato.

Fruit Phenotypes	Genetic Make up		
	*Psy1*	*SGR*	*SlMYB12*
Red	N	N	N
Yellow	M	N	N
Brown	N	M	N
Pink	N	N	M
Light yellow	M	N	M
Pink brown	N	M	M
Yellow green	M	M	N
Green	M	M	M

N—normal, and M—mutated.

**Table 2:** List of markers designed to follow the mutations in *Psy1*, *SGR*, and *SlMYB12* responsible for the eight fruit color phenotypes in the segregating F_2_ population with their respective PCR amplicon sizes and SNP positions in the sequences.

Gene	Primer Name	Sequence (5′-3′)	Fruit Phenotypes with Exact PCR Amplicon Size (bp)/SNP at bp							
			Red	Yellow	Brown	Pink	Light Yellow	Pink Brown	Yellow Green	Green
***Psy1***	Psy1CDSF	ATGTCTGTTGCCTTGTTATGGGTTGTTTC	396	468	396	396	468	396	468	468
	Psy1R5	TGCATACTCTGCACATACTTCACCAC								
***SGR***	SGR4-aR2	AGGATCTGACACAGGACCAATAACA	A at 1679	A at 1679	G at 1679	A at 1679	A at 1679	G at 1679	G at 1679	G at 1679
	SGR4-aF2	GAAGATGTCACTTCATGTCCATTG								
***SlMYB12***	aF1	GTGACGAACAACCGACCTAGAATAA	950 (aF1 + aR6) 614 (aF1 + aR5)	950 (aF1 + aR6) 614 (aF1 + aR5)	950 (aF1 + aR6) 614 (aF1 + aR5)	347 (aF1 + aR6)	347 (aF1 + aR6)	347 (aF1 + aR6)	950 (aF1 + aR6) 614 (aF1 + aR5)	347 (aF1 + aR6)
	aR6	GCGGACAAAGTTAATTGGTCACTCA								
	aR5	ATTCTAGCGTTATCAGTCGGCATACA								

**Table 3:** Component loading biochemical (carotenoids and chlorophylls) concentrations and mean PC scores of eight different fruit color phenotypes of tomato in the segregating F_2_ population as determined by principal component analysis (PCA).

Variable	PC1	PC2
Phytoene	0.385	0.002
Phytofluene	0.374	0.044
*cis*-lycopene	0.396	−0.002
*trans*-lycopene	0.408	0.000
γ-carotene	0.401	−0.010
β-carotene	0.353	−0.172
Lutein	0.267	−0.418
Chlorophyll a	−0.140	−0.622
Chlorophyll b	−0.115	−0.638
% variation explained	0.660	0.242
*p*-value	<0.01	<0.01
**Fruit Colour**	**Mean PC Sores**	
Red	3.59 a	0.86 a
Yellow	−1.97 cd	1.80 a
Brown	0.65 bc	−1.89 b
Pink	2.90 ab	0.69 a
Light yellow	−2.051 cd	1.88 a
Pink brown	1.12 ab	−1.17 b
Yellow green	−2.04 cd	−1.38 b
Green	−2.20 d	−0.79 b

## Supplementary Materials

Supplementary materials are available online. Figure S1: Confirmation of identified SNP of the *SGR* gene mutation using 3′-blocked and unlabeled oligonucleotide probe (Hybprobe). Figure S2: Schematic depiction of the *SlMYB12* gene illustrating the 603 bp deletion in the upstream region (a), Gel image showing 603 bp deletion in the upstream of *SlMYB12* gene in green fruited inbred line, BUC30 (b). Figure S3: Images of 8 different fruit colour tomato phenotypes in F_2_ population obtained from the cross between BUC30 (green fruit colour line) and KNR3 (red fruit colour line). Table S1: Primers and probes used in this study Table S2: Phenotypes of F2 population segregate for eight fruit colour of a cross between KNR3 × BUC30 with chi-squre test for goodness of fit Table S3: Respective Phenotype and genotype of F2 population of a cross between KNR3 × BUC30 Table S4: HPLC analysis of carotenoid and chlorophyll content in F2 population fruits Supplementary Data 1: Genomic, cds, and protein sequences of SGR gene for the genotypes Heinz1706, KNR3 (red fruited line), and BUC30 (green fruited line).
