# Supplementary material for: Molecular Insights Reveal Psy1, SGR, and SlMYB12 Genes are Associated with Diverse Fruit Color Pigments in Tomato (Solanum lycopersicum L.)
Source: Molecules. 2017 Dec 8;22(12):2180. doi: 10.3390/molecules22122180 (PMC6149895; doi:10.3390/molecules22122180)
Supplement: Supplementary file 1 [file molecules-22-02180-s001.zip › Supplementary materials/Supplementary figures.pptx]

## Slide 1
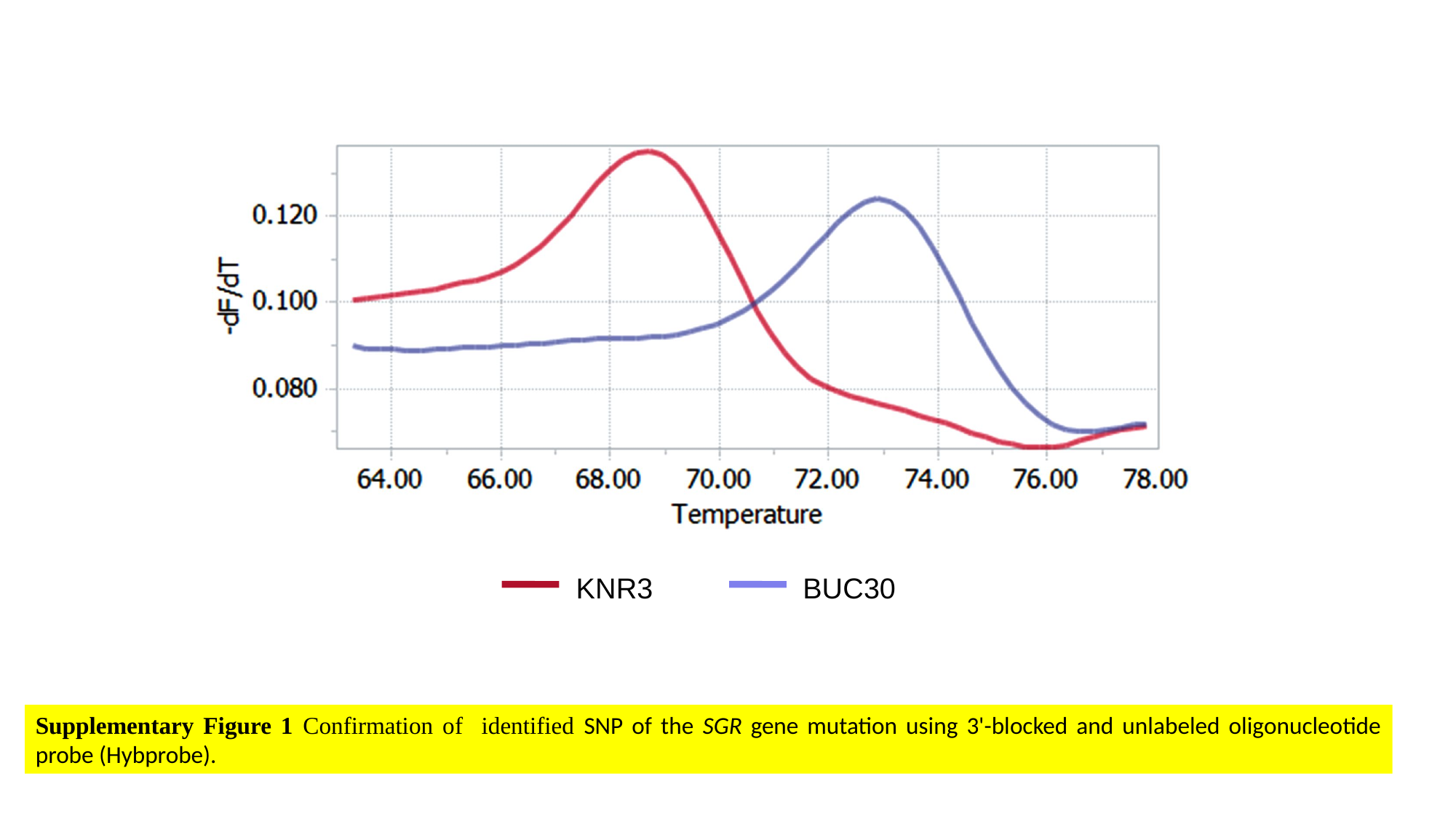

KNR3
BUC30
Supplementary Figure 1 Confirmation of identified SNP of the SGR gene mutation using 3'-blocked and unlabeled oligonucleotide probe (Hybprobe).

## Slide 2
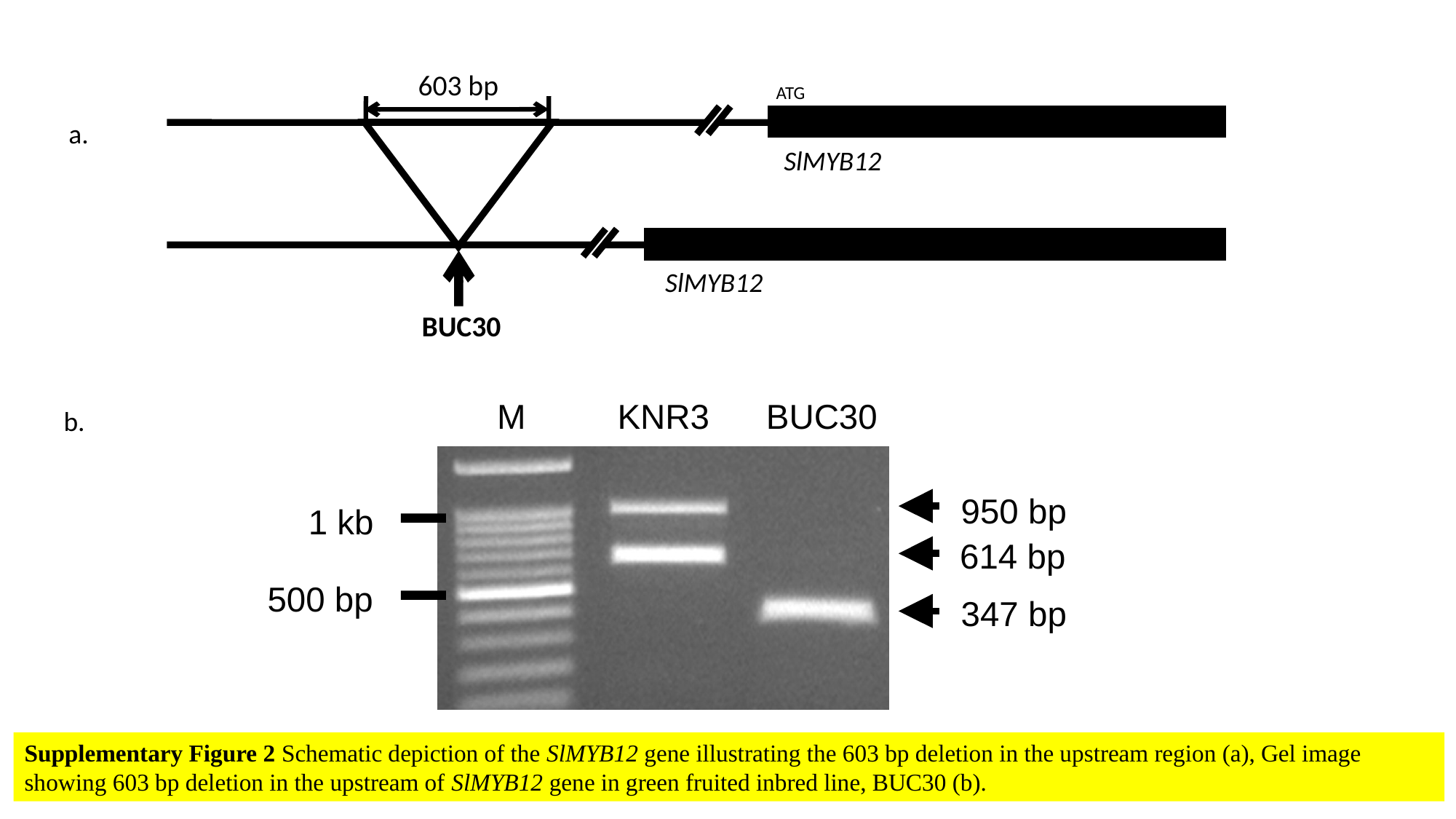

603 bp
ATG
SlMYB12
SlMYB12
BUC30
a.
M
KNR3
BUC30
950 bp
1 kb
614 bp
500 bp
347 bp
b.
Supplementary Figure 2 Schematic depiction of the SlMYB12 gene illustrating the 603 bp deletion in the upstream region (a), Gel image showing 603 bp deletion in the upstream of SlMYB12 gene in green fruited inbred line, BUC30 (b).

## Slide 3
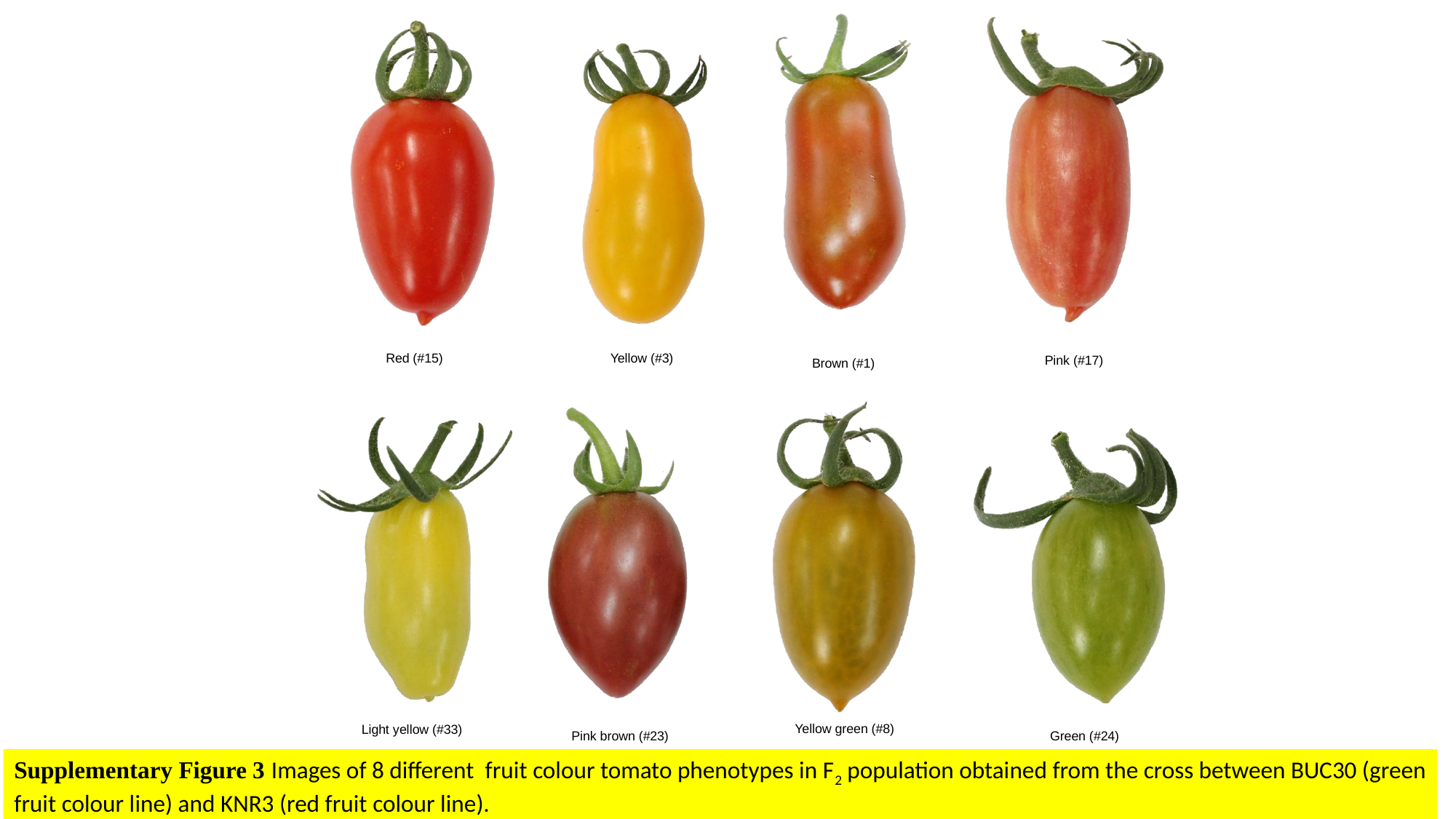

Red (#15)
Yellow (#3)
Pink (#17)
Brown (#1)
Yellow green (#8)
Light yellow (#33)
Pink brown (#23)
Green (#24)
Supplementary Figure 3 Images of 8 different fruit colour tomato phenotypes in F2 population obtained from the cross between BUC30 (green fruit colour line) and KNR3 (red fruit colour line).
